# Association of Macrophage Migration Inhibitory Factor‐794 CATT Microsatellite Polymorphism With Tuberculosis Risk: A Systematic Review and Meta‐Analysis

**DOI:** 10.1155/pm/8413702

**Published:** 2026-05-06

**Authors:** Anand Kumar Maurya, Sabir Ali, Shaina Gaikwad

**Affiliations:** ^1^ Department of Microbiology, All India Institute of Medical Sciences, Bhopal, Madhya Pradesh, India, aiims.edu; ^2^ Multidisciplinary Research Unit, All India Institute of Medical Sciences, Bhopal, Madhya Pradesh, India, aiims.edu

**Keywords:** CATT microsatellite polymorphism, cytokine gene regulation, genetic association study, macrophage migration inhibitory factor (MIF), tuberculosis susceptibility

## Abstract

**Background:**

Tuberculosis (TB) remains a major infectious cause of morbidity and mortality worldwide and is influenced by both environmental exposures and host genetic factors. Macrophage migration inhibitory factor (MIF), a proinflammatory cytokine, plays an important role in immune and inflammatory responses. The MIF‐794 CATT microsatellite polymorphism (rs5844572) in the promoter region may affect gene transcription and thereby influence susceptibility to TB. This systematic review and meta‐analysis are aimed at evaluating the association between the MIF‐794 CATT polymorphism and TB risk across different populations.

**Methods:**

A comprehensive literature search was performed in PubMed, Embase, Web of Science, Cochrane Library, and Google Scholar up to the latest available date. Case‐control studies evaluating the association between the MIF‐794 CATT polymorphism and TB susceptibility were included in accordance with PRISMA guidelines. Data from seven high‐quality studies (Newcastle–Ottawa Scale score ≥ 8), comprising 1063 TB cases and 957 controls, were pooled. Odds ratios (ORs) with 95% confidence intervals (CIs) were calculated under allelic, dominant, and recessive genetic models using fixed‐ or random‐effects models according to heterogeneity.

**Results:**

The pooled analysis suggested a possible association between longer CATT repeat alleles (CATT_7_ or CATT_8_) and increased TB susceptibility compared with shorter repeats (CATT_5_ or CATT_6_), although the effect size was modest and not fully consistent across all populations. A stronger trend was observed in some East Asian cohorts, whereas African and Latin American studies showed variable results. All included studies were of high methodological quality, and control groups were reported to be in Hardy–Weinberg equilibrium (HWE). Visual assessment of funnel plots did not indicate marked publication bias, although the small number of studies limits definitive interpretation.

**Conclusions:**

The available evidence suggests that the MIF‐794 CATT polymorphism may contribute to TB susceptibility, but the association remains modest, heterogeneous, and inconclusive overall. This variant should be regarded as a possible component of a broader immunogenetic framework rather than an established standalone biomarker. Further large‐scale, multicentric, and functionally integrated studies are needed to clarify its role in TB risk.

## 1. Introduction

Tuberculosis (TB) is a contagious infectious disease caused by *Mycobacterium tuberculosis* and characterized by persistent lung inflammation that can lead to tissue damage, loss of pulmonary function, and disability. Approximately 9 million people are affected by TB each year, making it a major global public health concern [1]. Infection with *M. tuberculosis* is mostly determined by the host immunological response, which is essential in deciding whether the infection is managed or develops into an active illness. When *M. tuberculosis* is detected, the innate immune system triggers an early antimycobacterial response [2], which affects the course of the infection and its long‐term management. The adaptive immune system′s presentation of antigens and the control of inflammation depend on this interaction [3]. However, *M. tuberculosis* can evade immune responses and exploit innate immune cells as sites of intracellular replication, thereby promoting chronic infection [4]. Although the immune system plays a crucial role in determining TB progression, genetic factors also influence an individual′s susceptibility to infection and disease severity. The immune system serves as the body′s first line of defense against TB. Genetic variations also contribute to differences in disease susceptibility and progression among individuals. A genetic component is known to play a role in TB infection and disease; for example, children with uncommon abnormalities in the IFN‐*γ*/IL‐12/IL‐23 axis are more susceptible to infection. It has been more challenging to pinpoint the role of genetics in adult infection [5, 6]. Variants in pattern recognition receptors DC‐SIGN, TLRs, NOD2, and LTHA4H, along with rare polymorphisms in SLC11A1 that influence phagosome function, are examples of candidate genes that impact macrophage handling of mycobacteria [6–8].

The occurrence and progression of TB are significantly influenced by polymorphisms in specific genes. Numerous genes linked to TB have been discovered in recent years [9–16]. A cytokine with proinflammatory and chemokine‐like properties, macrophage migration inhibitory factor (MIF) is essential for mediating a broad range of immune responses to invasive infections. Additionally, MIF might be linked to the development and/or course of TB. MIF, which limits macrophage movement, enhances macrophage accumulation, and activates T cells in inflamed TB lesions, is probably one of the earliest cytokines to emerge in the inflammatory response in TB [17, 18].

Two polymorphisms in the MIF promoter region have been well characterized: a microsatellite polymorphism‐794CATT_5–8_ and a single nucleotide polymorphism (SNP) at position −173 (G‐C) [19]. A repeat sequence of a functional microsatellite, 794 CATT_5–8_ (rs5844572), where an increase in the number of repeats corresponds to higher MIF expression; higher numbers of CATT repeats (CATT _6,7,8_) are linked to stronger promoter activity, whereas CATT_5_ is linked to low MIF expression [18, 20, 21]. Various studies have examined the effect of the MIF‐794 CATT_5–8_ gene polymorphism in TB risk, with varying degrees of success [22–27]. The −794 CATT_5_ variant corresponds to a low‐expression allele, whereas the 794CATT_6,7,8_ variants are associated with higher‐expression alleles. The number of CATT tetranucleotide repeats at position −794 is correlated with variants in MIF expression. Multiple studies have reported that the SNP at position −173 (G/C) in the MIF promoter is in linkage disequilibrium with the high‐expression, −794 CATT7 allele and is associated with increased susceptibility to disease [19]. The cytokine MIF has been implicated in the pathogenesis of various diseases. Moreover, polymorphisms in the human MIF promoter have been linked to the host′s susceptibility to infectious diseases [28]. Several recent studies have indicated that both of these polymorphisms are linked to TB susceptibility [24, 29].

This study is aimed at systematically evaluating the association between the functional MIF −794 CATT_5–8_ promoter microsatellite polymorphism and susceptibility to TB, including both new and retreatment cases. The objective was to clarify whether longer‐repeat alleles or genotypes are consistently associated with TB risk across different populations and to assess the strength and conclusiveness of the available evidence.

## 2. Materials and Methods

### 2.1. Protocol and Registration

This study was carried out as a systematic review and meta‐analysis to assess the association between the macrophage migration inhibitory factor‐794 (MIF‐794) CATT microsatellite polymorphism and susceptibility to TB. The research adhered to the Preferred Reporting Items for Systematic Reviews and Meta‐Analyses (PRISMA) guidelines to ensure methodological transparency, reproducibility, and reliability of the results. A detailed protocol was prospectively developed and registered in the International Prospective Register of Systematic Reviews (PROSPERO) (Identification Number CRD420251009985).

### 2.2. Literature Search Strategy

A comprehensive and systematic literature search was performed across multiple electronic databases, including PubMed, Embase, Web of Science, Cochrane Library, and Google Scholar, from database inception to the most recent available update. We used Boolean combinations of Medical Subject Headings (MeSH) and free‐text terms. A representative PubMed search strategy was: (“macrophage migration inhibitory factor” OR “MIF”) AND (CATT OR microsatellite OR “‐794”) AND (tuberculosis OR “pulmonary tuberculosis” OR TB) AND (polymorphism OR genotype OR “genetic association”). Equivalent search strings were adapted for the other databases using their specific syntax. Searches were restricted to articles published in the English language. In addition to database searches, we manually screened the reference lists of all eligible articles and relevant reviews to identify any additional potentially eligible studies.

### 2.3. Eligibility Criteria

Predetermined inclusion and exclusion criteria were applied to assess the eligibility of identified studies. Studies were included if they were cohort or case–control investigations assessing the relationship between TB susceptibility and the MIF‐794 CATT microsatellite polymorphism, were conducted on human participants with microbiologically and/or clinically confirmed TB and healthy or non‐TB controls, reported genotype and/or allele frequencies of the MIF‐794 CATT polymorphism, provided sufficient data to compute odds ratios (ORs) with 95% confidence intervals (CIs), and were published in the English language.

Studies were excluded if they were nonhuman studies (in vitro or animal experiments), nonoriginal articles such as review articles, conference abstracts, editorials, or case reports, did not specifically evaluate the MIF‐794 CATT polymorphism in relation to TB, lacked extractable genotype frequency data or other essential information for quantitative analysis, or represented duplicate publications, in which case only the most recent or the most comprehensive dataset was retained.

### 2.4. Data Extraction

Data extraction was performed independently by two reviewers using a predesigned standardized data collection form. From each eligible study, we recorded the following information:

• Study characteristics: first author, year of publication, country, study design, total sample size (number of TB cases and controls), and genotyping method used (e.g., TaqMan assay, PCR–DNA sequencing, or MS‐PCR).
• Participant characteristics: ethnicity or population background (e.g., Asian, African, and Latin American), mean or median age, distribution of sexes, clinical form of TB where available (pulmonary/extrapulmonary, new/retreatment), and diagnostic criteria used for TB confirmation.
• Genetic and outcome data: genotype and allele frequencies of the MIF‐794 CATT polymorphism in cases and controls, Hardy–Weinberg equilibrium (HWE) status in the control group, and reported or calculable ORs with 95% CIs under different genetic models.
• Quality indicators: Newcastle–Ottawa Scale (NOS) scores to assess methodological rigor.

A third reviewer was consulted in order to resolve any discrepancies in data extraction and to guarantee accuracy and consistency of the final dataset.

### 2.5. Quality Assessment

Three domains—participant selection, study group comparability, and exposure determination—were used to evaluate the quality of the included studies using the NOS. Each study was assigned a score ranging from 0 to 9. Studies scoring ≥ 7 were graded as high‐quality and included in the final quantitative synthesis.

### 2.6. Publication Bias Assessment

Potential publication bias was evaluated visually using Begg′s funnel plots. Symmetry in the funnel plots indicated the absence of major publication bias.

### 2.7. Expected Outcomes

The meta‐analysis is aimed at:

1. Determining the genetic association between *MIF-794 CATT microsatellite polymorphism* and TB susceptibility.
2. Identifying specific risk genotypes or alleles that may predispose individuals to TB.
3. Providing a consolidated evidence base to inform future molecular and epidemiological research on host genetic susceptibility to TB.

### 2.8. PRISMA Flow Summary

After a comprehensive systematic search, 395 potentially relevant records were identified through database searching (PubMed, Embase, Web of Science, Cochrane Library, and Google Scholar) and manual reference screening. Following the removal of 157 duplicates, 238 titles and abstracts were screened. Of these, 168 records were excluded because they were review articles, case reports, commentaries, or unrelated to the MIF‐794 CATT polymorphism.

The remaining 70 full‐text articles were thoroughly evaluated for eligibility. After detailed assessment, 63 studies were excluded for lacking data on MIF‐794 microsatellite polymorphisms or insufficient genotypic frequency information for quantitative synthesis. Finally, seven eligible case–control studies were included, encompassing a total of 1063 TB cases and 957 healthy controls.

### 2.9. Statistical Analysis

In order to evaluate the relationship between the MIF‐794 CATT polymorphism and TB risk, all statistical analyses were conducted to determine the pooled effect size (ORs with 95% CIs) under various genetic models.

• Allelic model: (CATT_7_ + CATT_8_ vs. CATT_5_ + CATT_6_).
• Dominant model: (CATT_7_/X + CATT_8_/X vs. CATT_5_/X + CATT_6_/X).
• Recessive model: (CATT_7_/CATT_7_ + CATT_8_/CATT_8_ vs. all other genotypes).

The I^2^ statistic and Cochran′s Q test were used to evaluate study heterogeneity. When heterogeneity was not significant (*I* ^2^ ≤ 50*%*, *p* ≥ 0.10), a fixed‐effects model was employed; when heterogeneity was substantial (*I* ^2^ > 50*%*, *p* < 0.10), a random‐effects model was utilized. Because the number of eligible studies was small and study‐level covariate, information was limited, formal meta‐regression and detailed subgroup analyses to explore sources of heterogeneity were not feasible. Therefore, heterogeneity was addressed primarily through model selection and sensitivity analysis.

A leave‐one‐out method was used to conduct sensitivity analyses in order to assess the stability of the combined results. Overall, leave‐one‐out sensitivity analyses for genotype‐ and allele‐based models showed that the pooled effect estimates were not unduly driven by any single study. However, these analyses were interpreted cautiously because moderate‐to‐high heterogeneity remained in some comparisons. (Details in Supporting files).

Trial sequential analysis (TSA) (two‐sided *α* = 0.05, power = 80*%*) was conducted for the allele comparison (7 + 8 vs. 5 + 6). Using the random‐effects pooled estimate (OR ≈ 1.19) as the anticipated effect and adjusting the required information size for between‐study heterogeneity (DARIS; I^2^ ≈ 72.5%), the diversity‐adjusted required information size was approximately 11,724 participants. The accrued evidence from included studies (*n* = 2023 participants; Kuai 2015 excluded due to missing allele counts) corresponded to ~17% of DARIS. The cumulative *Z*‐curve did not cross the O′Brien–Fleming trial sequential monitoring boundary (Z ≈ 3.58 vs. boundary ≈ 4.72), indicating that, despite a nominally significant conventional meta‐analytic result, the TSA remained inconclusive because the accrued information size was limited and the available evidence may still be vulnerable to random error. Accordingly, the TSA findings were interpreted as indicating that additional adequately powered studies are required before firm conclusions can be drawn. (Details in Supporting files).

Because one eligible study (Kuai 2015) did not report complete allele‐count data, it could not be included in pooled analyses requiring raw allele frequencies, including the allele‐based TSA. This exclusion was limited to analyses for which complete extractable allele‐count data were necessary.

Comprehensive Meta‐Analysis (CMA) Version 3.0 software was used for all statistical calculations, and the findings were displayed as forest plots to show effect sizes and CIs.

## 3. Results

### 3.1. Literature Search Results

This meta‐analysis ultimately included seven high‐quality case–control studies. The included studies were conducted in four distinct geographic regions and involved study populations from Brazil (South America), South Africa (sub‐Saharan Africa), China (East Asia), and Colombia (Latin America), providing a reasonably broad representation of population backgrounds and genetic diversity. The mean or median age of study participants ranged from 28 to 48 years, and both newly diagnosed as well as retreatment TB cases were represented, enhancing the generalizability of findings to real‐world clinical settings.

All included studies used standardized PCR‐based genotyping methods, including TaqMan SNP assays, microsatellite‐specific PCR followed by sequencing (MS‐PCR–Seq), and PCR followed by DNA sequencing to accurately detect variations in the MIF‐794 CATT microsatellite polymorphism. Importantly, all studies reported that genotype distributions in control groups were consistent with HWE, indicating the absence of major genotyping errors or substantial selection bias.

**Figure 1:**
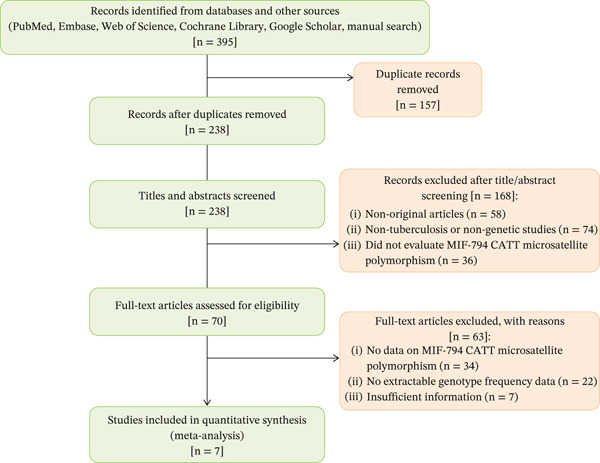
PRISMA flow diagram of study selection.

According to the NOS, the studies′ methodological quality ranged from 8 to 9, signifying a uniformly high standard of design, execution, and reporting. These studies were characterized by well‐defined inclusion criteria, appropriate control selection, and rigorous genotyping procedures. The study selection process is presented in Figure 1, and the main characteristics of the included studies are summarized in Table 1.

**Table 1:** Main characteristics of included studies in this meta‐analysis.

Author	Year	Country	Ethnicity (study population)	Genotyping methods	Mean age (*y* *e* *a* *r* ± median)		Sample size		HWE test	Quality score
					Cases	Controls	Cases	Controls		
Machado et al. [30]	2021	Brazil	American	PCR‐DNA seq	3 9.8 ± 15.5	32.3 ± 12.8	126	119	Y	8
Reid et al. [31]	2019	South Africa	African	MS‐PCR‐Seq and TaqMan SNP	DUR cohort 34 (IQR 29–38)	DUR cohort 34 (IQR 29–42)	101	95	Y	9
					TFG cohort	TFG cohort	100	65	Y	
					36 (IQR 31–46)	37 (IQR 28.5–46)				
Liu et al. [22]	2018	China	Asian	PCR‐DNA seq	Mean age ± SD	38.65 ± 9.15	200	100	Y	8
					TC − 40.56 ± 17.39					
					NW cases − 40.64 ± 20.24					
					RT cases − 0.52 ± 15.70					
Kuai et al. [21]	2015	China	Asian	TM	48.4 ± 10.5	48.4 ± 10.5	47	50	Y	9
Li et al. [24]	2012	China	Asian	TM	43.8 ± 5.76	40.2 ± 4.9	215	245	Y	8
Li et al. [25]	2012	China	Asian	TM	47.8 ± 5.76	40.2 ± 4.9	151	149	Y	8
Gomez et al. [18]	2007	Colombia	Latin American	TM	40 ± 16	43 ± 16	223	134	Y	8

Abbreviations: DUR, Durban cohort; MS‐PCR‐Seq & TaqMan SNP, microsatellite PCR‐sequencing with TaqMan SNP assay; NW, new cases; PCR‐DNA seq, PCR followed by DNA sequencing; RT, retreatment; TC, total cases; TFG, Tugela ferry/Greytown cohort; TM, TaqMan.

### 3.2. Genotypic Distribution of MIF‐794 CATT Polymorphism

**Figure 2:**
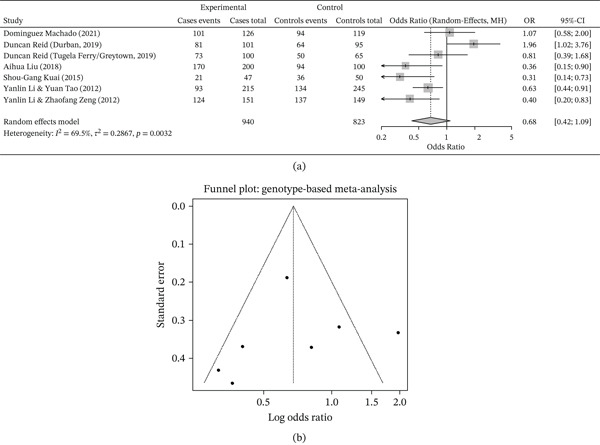
(a) Distribution of genotypes of MIF‐794 CATT in included studies. (b) Funnel plot of studies included for the genotype comparison of MIF‐794 CATT.

The genotypic frequency distribution of the MIF‐794 CATT polymorphism among TB cases and healthy controls is summarized in Table 2 and visualized in Figure 2 a. Across most studies, the low‐repeat genotypes (5/*X* + 6/*X*) were more prevalent than the high‐repeat genotypes (7/*X* + 8/*X*) in both cases and controls. When comparing proportions across individual cohorts, TB cases tended to show a higher frequency of high‐repeat (≥ 7) genotypes in several studies, although some cohorts demonstrated comparable or even higher frequencies in controls, reflecting interpopulation heterogeneity.

**Table 2:** Distribution of alleles and genotypes of MIF‐794 CATT in included studies in this meta‐analysis.

Author	Year	Sample size		Genotypes				Alleles			
				Cases		Controls		Cases		Controls	
		Cases	Controls	5/*X* + 6/*X*	7/*X* + 8/*X*	5/*X* + 6/*X*	7/*X* + 8/*X*	5 + 6	7 + 8	5 + 6	7 + 8
Machado et al. [30]	2021	126	119	101	25	94	25	221	31	205	33
Reid et al. (DUR) [31]	2019	101	95	81	17	64	30	178	18	156	32
Reid et al. (TFG) [31]		100	65	73	27	50	14	172	28	110	18
Liu et al. [22]	2018	200	100	170	30	94	6	315	85	175	25
Kuai et al. [21]	2015	47	50	21	26	36	14	NA	NA	NA	NA
Li et al. [24]	2012	215	245	93	122	134	111	266	164	363	127
Li et al. [25]	2012	151	149	124	27	137	12	195	107	222	76
Gomez et al. [18]	2007	223	134	NA	NA	NA	NA	368	78	223	46

*Note:* Allele counts were unavailable for Kuai 2015; therefore, this study was included in genotype‐based analysis where possible but excluded from allele‐based synthesis and trial sequential analysis. Missing values are shown as NA.

For instance, Machado et al. observed that among 126 Brazilian TB cases, 25 (19.8%) carried the high‐repeat genotype compared with 25 (21.0%) among 119 controls [30], indicating broadly similar carriage of high‐repeat genotypes between cases and controls in that population. In the DUR cohort of Reid et al., 17 of 101 TB cases (16.8%) harbored high‐repeat alleles compared with 30 of 95 controls (31.6%), whereas in the TFG cohort, 27 of 100 cases (27%) had 7/8 repeats compared with 14 of 65 controls (21.5%) [31], highlighting that different South African cohorts can show divergent patterns. Among the Chinese cohorts, Liu et al. reported high‐repeat genotypes in 15% of cases versus 6% of controls [22], and Li et al. (2012) noted similar findings with 38% versus 31% [24]. Taken together, these observations point to population‐level differences but suggest, particularly in East Asian cohorts, a tendency towards higher frequencies of 7/*X* + 8/*X* genotypes among TB cases.

Overall, these genotype‐based findings indicate a possible association between higher‐repeat genotypes and TB susceptibility in some populations, but the pattern was not uniform across all included cohorts. Therefore, the descriptive genotype distributions should be interpreted cautiously and in the context of between‐study heterogeneity.

The funnel plot for genotype‐based comparisons (Figure 2) b demonstrated no obvious marked asymmetry on visual inspection, suggesting no clear evidence of publication bias, although the limited number of studies requires cautious interpretation.

### 3.3. Allelic Distribution of MIF‐794 CATT Polymorphism

**Figure 3:**
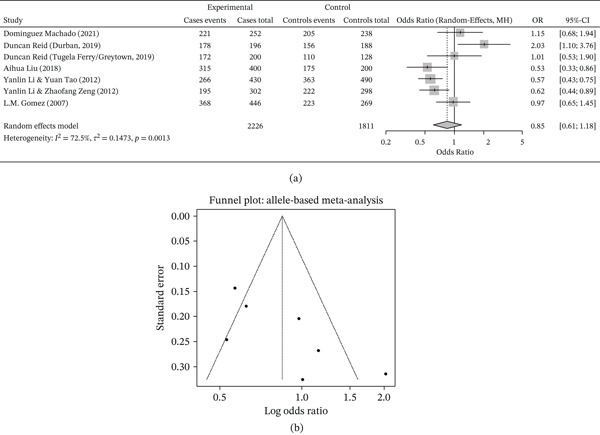
(a) Distribution of alleles of MIF‐794 CATT in included studies. (b) Funnel plot of studies included for the allele comparison of MIF‐794 CATT.

The allelic frequency distribution across studies (Table 2; Figure 3) a further supports the genotypic findings. In most included cohorts, the CATT_5_ and CATT_6_ (short‐repeat) alleles were predominant; however, CATT_7_ and CATT_8_ (long‐repeat) alleles appeared relatively enriched among TB patients. For example, Machado et al. observed allelic counts of 221 (CATT_5 + 6_) and 31 (CATT_7 + 8_) among cases, compared with 205 and 33 among controls, respectively [30]. Liu et al. reported 315 (CATT_5 + 6_) versus 85 (CATT_7 + 8_) alleles in TB cases and 175 versus 25 among controls, highlighting a greater proportion of long repeats among cases [22]. Similarly, Li et al. found an elevated proportion of high‐repeat alleles (127 vs. 164) among TB patients compared with controls [24].

However, not all included studies provided allele‐count data in a form suitable for all pooled allele‐based analyses. In particular, one study could not be incorporated into specific allele‐based comparisons because complete extractable allele‐frequency data were unavailable. Therefore, the number of studies contributing to some allelic analyses was smaller than the total number of eligible studies, and these results should be interpreted accordingly.

The overall trend suggests that long‐repeat (≥ 7) alleles are associated with increased TB susceptibility, although this association was modest and not fully consistent across all included populations. Thus, the allelic findings are more appropriately interpreted as suggestive of a possible risk‐increasing effect rather than definitive evidence of a uniform association. The funnel plot for allele‐based comparisons (Figure 3) b also showed an approximately symmetrical distribution, indicating a low apparent likelihood of major publication bias in allele‐level analyses, although this visual assessment is limited by the small number of studies.

Integration of genotypic and allelic data (Tables 1 and 2; Figures 2 and 3) across multiethnic cohorts revealed a broadly consistent pattern: individuals carrying higher MIF‐794 CATT repeats (seven or eight) tended to have a moderately higher risk of TB compared with those carrying shorter repeats (five or six), particularly in East Asian populations.

Despite some variation in effect sizes between geographic regions and individual cohorts, the overall evidence from high‐quality studies supports a positive association between long‐repeat alleles and TB risk. Rather than a single universal effect, the data suggest a context‐dependent impact of MIF‐794 CATT length that is modulated by population background and possibly by environmental cofactors. Funnel plots (Figures 2b and 3b) did not reveal marked publication bias, further supporting the reliability of the meta‐analytic estimates while still acknowledging the limitations imposed by the relatively small number of available studies.

Taken together, the results support a possible population‐dependent relationship between longer MIF‐794 CATT repeats and TB susceptibility, but the observed heterogeneity and incomplete allele‐level reporting in some studies warrant cautious interpretation of the pooled findings.

## 4. Discussion

This systematic review and meta‐analysis synthesizes evidence from seven high‐quality case–control studies conducted in Brazil, South Africa, China, and Colombia to examine whether the MIF‐794 CATT (rs5844572) promoter microsatellite polymorphism is associated with susceptibility to TB. Overall, the pooled findings suggest a possible association between longer repeat alleles (CATT_7_ or CATT_8_) and increased TB risk; however, the strength and statistical significance of this relationship were not uniform across studies or populations. Although several comparisons showed a risk‐increasing trend, the observed variability indicates that the evidence should be interpreted cautiously and does not support a universally consistent effect across all settings.

One notable pattern in the included literature is that the association appeared more evident in some East Asian cohorts, where longer repeat alleles or genotypes were reported more frequently among TB cases than controls. Liu et al. provided supportive evidence by combining genetic association data with functional observations, showing that CATT_7/8_ genotypes and the CATT_8_ allele were more frequent among TB patients and were also associated with higher circulating MIF concentrations [22]. These findings are biologically relevant because they suggest that repeat‐length variation may influence host inflammatory responses. However, even with this functional support, the inference remains probabilistic rather than definitive, as genetic association does not establish causality and the number of available studies remains limited. Liu et al. also reported similar patterns in both newly diagnosed and retreatment TB, which may indicate that the variant is more closely related to host susceptibility than to treatment history alone [22].

In contrast, not all included studies followed the same direction. The Brazilian cohort reported by Machado et al. showed a partially discordant pattern, in which the CATT_5/5_ genotype and the CATT_5_ allele were more frequent among TB cases, whereas high‐repeat genotypes did not differ significantly between cases and controls [30]. This inconsistency is important because it cautions against overly generalized conclusions. Differences in ancestry, local linkage disequilibrium patterns, environmental exposures, comorbid conditions, or other unmeasured modifiers may influence the observed relationship between promoter repeat length and disease risk. These explanations remain hypothetical in the absence of direct functional confirmation in those populations, but the Machado et al. findings clearly indicate that the effect of MIF‐794 CATT variation is unlikely to be uniform across populations [30].

Evidence from other Chinese studies also supports the possibility of a risk‐increasing effect of longer repeats. Li et al. (2012) reported higher frequencies of CATT_7_ and CATT_8_ alleles among TB patients and also provided functional evidence linking increased repeat number with greater promoter activity and gene expression [24]. These data are consistent with the idea that promoter microsatellite length may modulate inflammatory signaling thresholds relevant to TB pathogenesis. Similarly, Ma et al., in an earlier meta‐analysis, reported significant associations in allelic and genotype‐based models and concluded that longer repeats increased TB risk [23]. The present synthesis extends that body of evidence by incorporating additional studies from multiple regions, including South Africa and Brazil. At the same time, the broader geographic coverage in the current review also makes the between‐study variability more apparent, suggesting that any pooled effect is likely modest and context dependent rather than universally stable across ethnic groups and epidemiological settings [23, 30, 31].

The South African cohorts reported by Reid et al. further illustrate this complexity. The Durban and Tugela Ferry/Greyton cohorts showed differing genotype and allele distributions, as well as variation in effect estimates, implying that even within one country, genetic associations may differ according to cohort composition and local context [31]. Factors such as ancestry structure, HIV prevalence, exposure burden, recruitment approach, and clinical definitions may all contribute to these differences. This within‐country variability reinforces the importance of cautious interpretation and supports the use of random‐effects approaches when synthesizing evidence across heterogeneous populations.

Heterogeneity remains an important consideration in interpreting these findings. Differences in allele frequencies, study design, sample size, genotyping methods, and population structure may partly explain the variability observed between studies. In addition, TB susceptibility is influenced by multiple interacting factors, including environmental exposure, comorbidities, and pathogen diversity, which may modify host genetic effects. Because only a small number of studies were available and study‐level covariate, information was limited, formal exploration of heterogeneity through meta‐regression or subgroup analysis was not feasible. Therefore, the observed association should be interpreted as suggestive rather than definitive.

Another important point is the interpretation of TSA. Although some pooled comparisons suggested a risk‐increasing trend for longer repeat alleles, the TSA findings do not support a firm conclusion at present. The accrued information size remained limited, and the cumulative *Z*‐curve did not provide definitive evidence sufficient to confirm a conclusive association. Therefore, the TSA results should be interpreted as indicating that the current evidence base is still underpowered and that additional well‐designed studies are needed before robust inferences can be made.

Because MIF is involved in upstream immune regulation and cytokine signaling, higher‐expression variants could plausibly influence host responses during *M. tuberculosis* infection [32, 33]. From a biological perspective, the observed trends are compatible with previous functional evidence showing that increasing −794 CATT repeat number can enhance promoter activity and MIF expression [20, 24, 34]. Nonetheless, mechanistic interpretation should remain conservative. Greater inflammatory activity may be beneficial in some contexts and harmful in others, and promoter variation is unlikely to determine disease susceptibility independently. Rather, it may contribute to one component of a broader host‐response profile that interacts with other genetic, environmental, and microbial factors.

The translational relevance of these findings remains limited at present. Although the MIF‐794 CATT polymorphism may contribute to TB susceptibility in some populations, current evidence does not support its use as an independent clinical biomarker. Instead, it may represent one component of a broader immunogenetic framework that warrants further investigation through studies integrating genetic, functional, and immunological data.

Several limitations should be acknowledged. First, the total number of eligible studies was small, reducing the precision of pooled estimates and limiting the ability to explore subgroup effects in depth. Second, not all studies reported data in a sufficiently detailed or standardized manner, particularly for genotype categories, allele distributions, and population‐level modifiers, which constrained secondary analyses. In particular, one study did not provide complete allele‐count data in a form suitable for all pooled allele‐based analyses, which limited its inclusion in specific comparisons and should be considered when interpreting those results. Third, residual confounding and population stratification cannot be excluded in observational case–control studies. Fourth, gene–gene and gene–environment interactions could not be adequately evaluated, despite their likely importance in TB susceptibility. Accordingly, the results of this review should be interpreted as hypothesis‐supporting rather than definitive.

In summary, the current meta‐analysis suggests that the MIF‐794 CATT promoter microsatellite polymorphism may be associated with TB susceptibility, with several studies indicating a tendency for longer repeats (CATT_7/8_) to be linked to higher risk, particularly in some East Asian cohorts [22, 24, 30, 31]. This interpretation is biologically plausible and supported by functional evidence connecting repeat length with promoter activity and inflammatory regulation [20, 34]. However, the overall evidence remains inconclusive, as the observed effect is modest, heterogeneous, and population dependent, and TSA did not demonstrate firm evidence. Therefore, MIF‐794 CATT variation should currently be regarded as a possible contributory factor rather than a definitive or uniform genetic determinant of TB susceptibility.

## 5. Conclusion

This systematic review and meta‐analysis provide updated evidence on the relationship between the MIF‐794 CATT microsatellite polymorphism and susceptibility to TB. Across seven high‐quality case–control studies from diverse populations, the pooled findings suggest a possible tendency for longer repeat alleles (CATT_7_ or CATT_8_) to be associated with increased TB risk. However, the observed effect was modest, heterogeneous across populations, and not consistently significant across all comparisons.

The biological plausibility of this association is supported by prior evidence indicating that longer −794 CATT repeats may increase MIF promoter activity and cytokine expression, which could influence inflammatory responses during *M. tuberculosis* infection. Nevertheless, the current evidence does not establish this polymorphism as an independent or clinically actionable predictor of TB susceptibility. In addition, TSA indicates that the currently available evidence remains inconclusive, as the accrued information size is limited and firm conclusions cannot yet be drawn.

In conclusion, the MIF‐794 CATT polymorphism may represent one component of a broader immunogenetic framework influencing TB susceptibility, but current evidence remains insufficient to support strong or universal inferences. Further large‐scale, multicentric studies involving diverse populations, standardized genetic reporting, complete genotype and allele data, and functional validation are needed to better define the magnitude, consistency, and clinical relevance of this association.

## Author Contributions

Conceptualization: A.K.M. and S.A.; methodology: A.K.M. and S.A.; software: S.A.; validation: A.K.M. and S.A.; database searching: S.A. and S.G.; report screening: S.A. and S.G.; quality: A.K.M. and S.A.; data extraction and analysis: S.A. and S.G.; writing—original draft preparation: S.A. and S.G.; writing—review and editing: A.K.M., S.A., and S.G.; visualization: S.A. and S.G.; supervision: A.K.M. and S.A.; project administration: A.K.M.; A.K.M., S.A., and S.G. contributed equally.

## Funding

No funding was received for this manuscript.

## Disclosure

All authors have read and agreed to the published version of the manuscript.

## Ethics Statement

Our comprehensive protocol was developed and submitted prospectively to the International Prospective Register of Systematic Reviews (PROSPERO) (CRD420251009985).

## Conflicts of Interest

The authors declare no conflicts of interest.

## Data Availability

The data from this study can be obtained from the corresponding author upon reasonable request.
